# Accuracy of Artificial Intelligence in Diagnosing Obstructive Sleep Apnea Using Photoplethysmography: Systematic Review and Meta-Analysis

**DOI:** 10.2196/78718

**Published:** 2026-09-25

**Authors:** Brian Sheng Yep Yeo, Esther Yanxin Gao, Joan Ern Xin Tan, Jun Yuan Koo, Yi Siang Lee, Nicole Kye Wen Tan, Adele Chin Wei Ng, Zhou Hao Leong, Thun How Ong, Leong Chai Leow, Guang-Bin Huang, Benjamin Kye Jyn Tan, Song Tar Toh

**Affiliations:** 1 Yong Loo Lin School of Medicine, National University of Singapore, 10 Medical Dr, Singapore, 117597, Singapore, 65 65161048; 2 Department of Otorhinolaryngology – Head and Neck Surgery, Singapore General Hospital, Outram Road, Singapore, 169608, Singapore, +6563214790; 3 Duke-NUS Sleep Centre, SingHealth, Singapore, Singapore; 4 Department of Respiratory and Critical Care Medicine, Singapore General Hospital, Singapore, Singapore; 5 School of Automation, Southeast University, Nanjing, China

**Keywords:** obstructive sleep apnea, artificial intelligence, AI, photoplethysmography, polysomnography, Bayesian statistics, diagnostic test accuracy

## Abstract

**Background:**

The traditional method for diagnosing obstructive sleep apnea (OSA) through polysomnography may be expensive and inaccessible. Recent developments in AI propose the use of photoplethysmography (PPG) to aid OSA diagnosis.

**Objective:**

This study seeks to evaluate the diagnostic accuracy of an AI-based approach in diagnosing OSA using PPG.

**Methods:**

PubMed, Embase, Scopus, Web of Science, and IEEE Xplore were searched from inception to November 3, 2025. The inclusion criteria comprised observational studies that evaluated the accuracy of AI-based methods of OSA diagnosis using PPG compared with conventional sleep testing used in adults. We excluded case reports, case series, reviews, meta-analyses, letters, conference abstracts, pediatric studies, animal studies, foreign language studies, studies with incomplete data, and studies focusing on individual apneic events without patient-level classification. The outcome of interest was the diagnostic accuracy of PPG-based AI models for OSA, as assessed in primary studies using random split tests, cross-validation, or external validation. Independent reviewers extracted data and assessed risk of bias using the QUADAS-2 (Quality Assessment of Diagnostic Accuracy Studies-2) tool. A Bayesian bivariate meta-analysis was used to pool estimates. Further subgroup, sensitivity, and meta-regression analyses were conducted. Overall quality of evidence was assessed using the GRADE (Grading of Recommendations, Assessment, Development and Evaluations) framework.

**Results:**

From 12,579 records, we included 13 studies, comprising 9983 participants. All studies were rated as either low or unclear for risk of bias. The overall evidence quality was moderate. AI trained on PPG achieved a pooled sensitivity of 79.6% (95% credible interval [CrI] 55.5%‐93.8%) and specificity of 76.5% (95% CrI 48.2%‐94.0%), compared to conventional diagnosis. The overall specificity increased (apnea-hypopnea index [AHI] ≥5: 63.6%; AHI ≥15: 81.8%; AHI ≥30: 85.1%), but overall sensitivity decreased (AHI ≥5: 87.2%; AHI ≥15: 79.7%; AHI ≥30: 76.7%) with greater categorical AHI severity cutoffs. Additionally, deep learning models achieved a higher specificity (82.9%) than traditional machine learning (63.6%). OSA prevalence and device type were not clearly associated with sensitivity or specificity.

**Conclusions:**

AI models trained on PPG have reasonable accuracy and may potentially serve as a low-cost screening tool. However, limitations such as small sample sizes, potential sources of bias, underrepresentation of certain geographic regions, and the influence of potential confounding factors highlight the necessity for further research. Future work should focus on deep learning to improve the feasibility and accessibility of this approach in primary care.

## Introduction

Obstructive sleep apnea (OSA) is a debilitating sleep-breathing disorder defined by recurring episodes of partial or complete upper airway collapse during sleep. This results in reduced or absent airflow lasting for at least 10 seconds and is associated with either cortical arousal or a decline in blood oxygen saturation [1]. It is estimated to affect 1 billion individuals globally, highlighting the need for effective and cost-efficient diagnostic and treatment approaches to reduce its adverse health sequelae [2]. OSA may be diagnosed with either home-based or laboratory-based sleep testing, with overnight polysomnography (PSG) touted as its gold standard diagnostic tool [3,4]. While it provides reliable diagnostic accuracy, it is constrained by high operating costs, limited accessibility in outpatient settings, patient inconvenience, and significant logistical and labor-intensive demands [5].

AI has emerged as a promising tool in medical diagnostics, leveraging machine learning algorithms to analyze complex clinical data and support diagnostic decision-making [6,7]. AI models are increasingly being assessed as automated alternatives to traditional PSG for OSA diagnosis. While AI-based approaches for OSA diagnosis have been explored using various data sources [8-11], the diagnostic performance of AI models trained on photoplethysmography (PPG) has not been systematically evaluated. PPG is a noninvasive, cost-effective, and portable optical technique used to provide real-time measurements of blood oxygen saturation in the microvascular tissue bed [12]. Presently, preexisting studies differ in opinion on the accuracy of AI-based diagnosis of OSA using PPG [13,14].

Given the growing presence of AI in medical diagnostics amid a digital revolution, and the expanding body of evidence, it is both clinically pertinent and timely to conduct a comprehensive synthesis of the evidence regarding the utility of AI models for OSA diagnosis, specifically those trained using PPG data [15]. The aim of this meta-analysis is to assess the diagnostic accuracy of AI models in detecting OSA using PPG.

## Methods

This systematic review and meta-analysis was registered on PROSPERO (CRD42024534235) and adhered to PRISMA (Preferred Reporting Items for Systematic Reviews and Meta-Analyses) guidelines. The PRISMA checklist can be found in Checklist 1 [16].

### Search Strategy

We conducted a comprehensive search of PubMed, Embase, Scopus, Web of Science, and IEEE Xplore from inception to November 3, 2025, using search terms pertaining to OSA, AI, PPG, and their relevant synonyms. The full search strategy is provided in Supplementary Methods in Multimedia Appendix 1.

### Study Selection

The study selection process by titles and abstracts, followed by full-text articles, was conducted by 6 independent authors (BSYY, EYG, JEXT, JYK, NKWT, BKJT), with disagreements resolved through the consensus of another independent author (STT). We selected observational studies involving adults aged 18 years or older, focused on the diagnosis or classification of OSA using AI, including both traditional and deep learning models, using PPG. The comparators included the diagnosis and classification of OSA based on the apnea-hypopnea index (AHI) derived from established methods of testing, including PSG or home sleep apnea testing (HSAT). The outcome of interest was the diagnostic accuracy of AI-based OSA diagnosis, evaluated using a random split test, cross-validation, or external validation. Table S1 in Multimedia Appendix 1 summarizes the inclusion criteria of the study. We excluded case reports, case series, reviews, meta-analyses, letters, conference abstracts, pediatric studies, animal studies, foreign language studies, studies with incomplete data, and studies focusing on individual apneic events without patient-level classification. Table S2 in Multimedia Appendix 1 summarizes the exclusion criteria.

### Data Extraction

The data of interest from the included articles were extracted by 3 independent authors (JEXT, JYK, YSL) into a structured pro forma. The details of data extraction were verified by independent authors (BSYY, BKJT). This included information pertaining to the first author, year published, country, percentage of males, mean age, mean body mass index, sample size, type of sleep study, reference AHI category thresholds, categorical AHI cutoffs, OSA prevalence, test method, AI model type, and type of sleep device used. Information regarding diagnostic accuracy, including measures of accuracy, sensitivity, specificity, and prevalence, was also extracted for analysis.

### Statistical Analysis

We conducted a Bayesian bivariate random-effects meta-analysis to jointly estimate sensitivity and specificity on the logit scale, accounting for their correlation [17-20]. To incorporate multiple AI models and AHI thresholds reported within the same study, we specified a hierarchical model with 2 levels, where study-level random effects captured between-study heterogeneity in diagnostic performance, while model-level random effects (nested within studies) captured variation across models and thresholds within the same study. Random effects at both levels were assumed to follow bivariate normal distributions, allowing sensitivity and specificity to be correlated. This approach accounted for the trade-off between sensitivity and specificity across and within studies. Pooled sensitivity and specificity were presented through summary receiver operating characteristic (SROC) curves. We performed meta-regression analyses by including study- and model-level covariates as fixed effects on the logit scale.

The following factors were examined: (1) OSA severity stratified by categorical AHI thresholds, with AHI ≥5, ≥15, and ≥30 corresponding to mild, moderate, and severe disease, respectively; (2) AI model type, distinguishing deep learning from traditional machine learning approaches; (3) type of primary validation method, including external validation using independent cohorts, cross-validation, or random data splitting; (4) OSA prevalence; and (5) type of sleep device, differentiating wearable smart devices from clinical oximeters. All results are reported as posterior means with 95% credible intervals (CrIs).

To evaluate for potential publication bias, Deeks test for funnel plot asymmetry was performed under varying probabilities for unpublished studies [21]. Further sensitivity analyses were conducted by recalculating diagnostic accuracy outcomes under each scenario using a Bayesian hierarchical framework, where an informative prior was applied with a lower bound set at 50% for both sensitivity and specificity.

### Risk of Bias Assessment

The QUADAS-2 (Quality Assessment of Diagnostic Accuracy Studies-2) tool was employed to evaluate the risk of bias and the applicability of the included diagnostic accuracy studies [22]. This tool allows for a structured assessment of potential biases and relevance of the studies using 4 domains: patient selection, index test, reference standard, and flow and timing. Three independent authors (JEXT, JYK, YSL) assessed each study, categorizing the risk of bias and applicability concerns as low, high, or unclear. Disagreements from the risk of bias assessment were resolved by independent authors (BSYY, BKJT).

### Overall Quality of Evidence

The overall quality of pooled evidence at the outcome level was assessed using the GRADE (Grading of Recommendations, Assessment, Development and Evaluations) framework [23,24]. This framework evaluates the quality of evidence by considering factors such as study design, consistency of results, directness of evidence, risk of bias, precision of estimates, and potential publication bias. The level of evidence for each outcome is categorized as high, moderate, low, or very low.

## Results

### Study Selection

**Figure 1.**
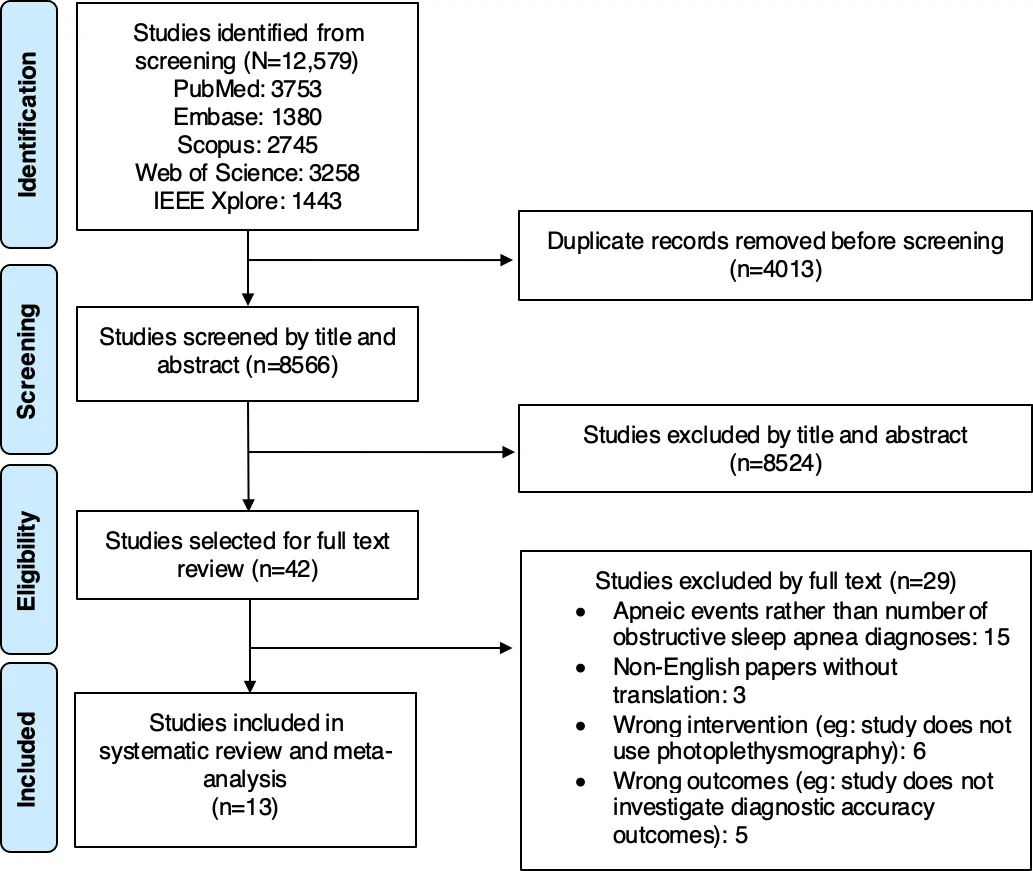
PRISMA (Preferred Reporting Items for Systematic Reviews and Meta-Analyses) flowchart for identification of studies via databases and registers.

A PRISMA flowchart documenting the study selection process is outlined in Figure 1. From a comprehensive search of 12,579 articles from PubMed, Embase, Scopus, Web of Science, and IEEE Xplore, 8566 nonduplicated records were screened at the title and abstract stage. Subsequently, 42 studies were screened for full-text eligibility, and 13 studies were included and qualitatively analyzed, comprising 9983 participants [13,14,25-35].

### Study Characteristics

Baseline study characteristics of included articles are found in Table S3 in Multimedia Appendix 1. All studies were cross-sectional in nature, and all studies used data from patients recruited from outpatient sleep clinics. The mean age of patients ranged from 42.0 to 69.4 years, and the percentage of male participants ranged from 46.4% to 74.0%. There were 5 studies from China, 2 from the United States, and 1 each from the United Kingdom, the Netherlands, Israel, Taiwan, South Korea, and Thailand. Using the QUADAS-2 scale, the risk of bias was listed as low for 7 studies and unclear for 6 studies (Table S4 in Multimedia Appendix 1).

### Diagnosis of OSA

All studies used PSG as the reference standard for diagnosing OSA, except for 1 study that used HSAT. The AHI served as the primary criterion for classification. Different studies used varying AHI thresholds, with ≥5, ≥15, and ≥30 events per hour representing mild, moderate, and severe OSA, respectively.

### Evaluation Methods and AI Models

Based on the primary validation strategy, 4 studies used random split, 3 studies adopted cross-validation, 5 studies conducted external validation, and 1 study used cross-validation and external validation for separate data cohorts. There were 7 studies that used deep learning and 6 studies that used traditional machine learning. Table S3 in Multimedia Appendix 1 outlines specific model subtypes.

### Overall Diagnostic Accuracy

**Figure 2.**
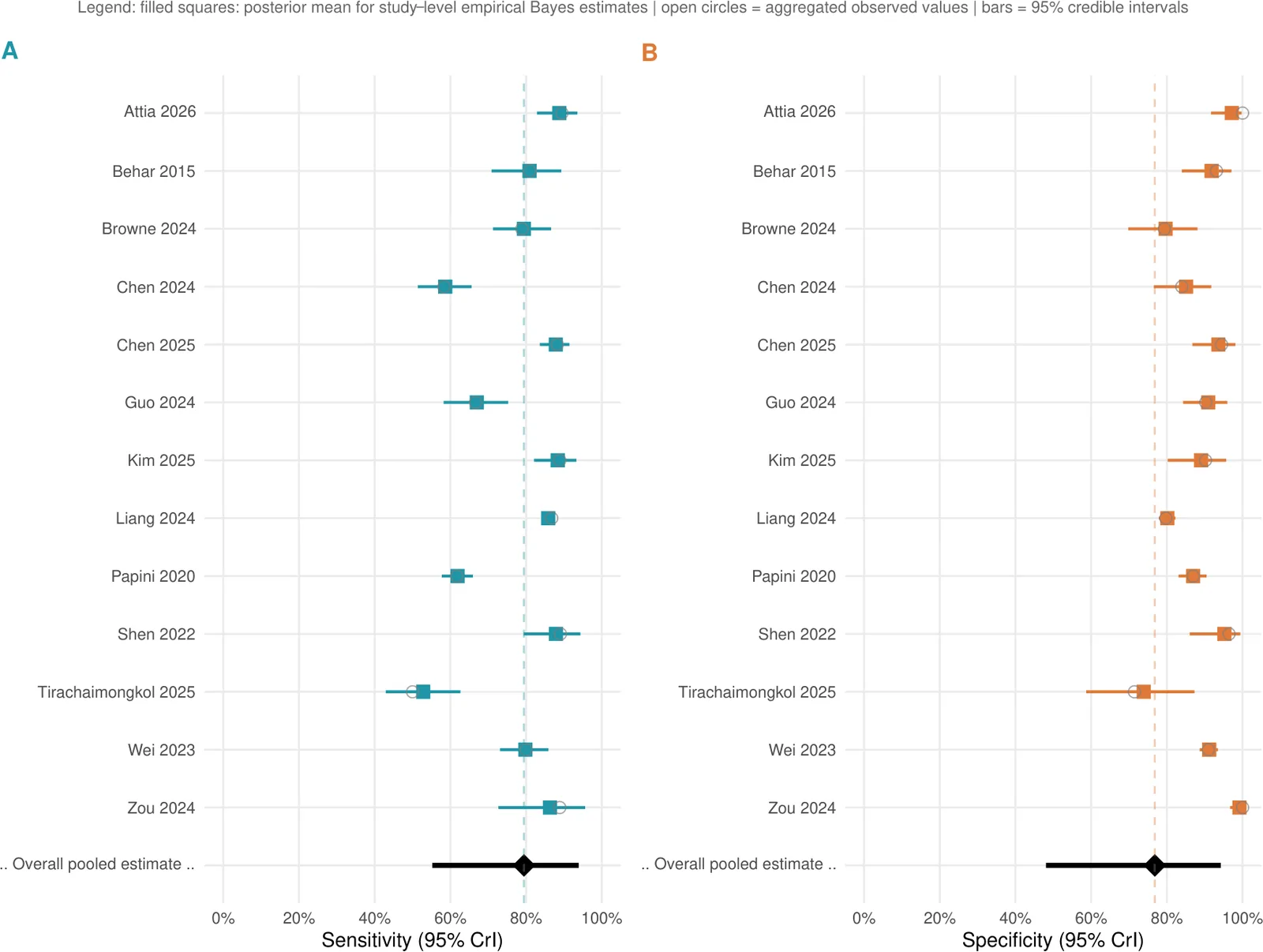
Bayesian bivariate meta-analysis of photoplethysmography-based obstructive sleep apnea detection. Results for (A) sensitivity and (B) specificity [13,14,25-35]. CrI: credible interval.

**Figure 3.**
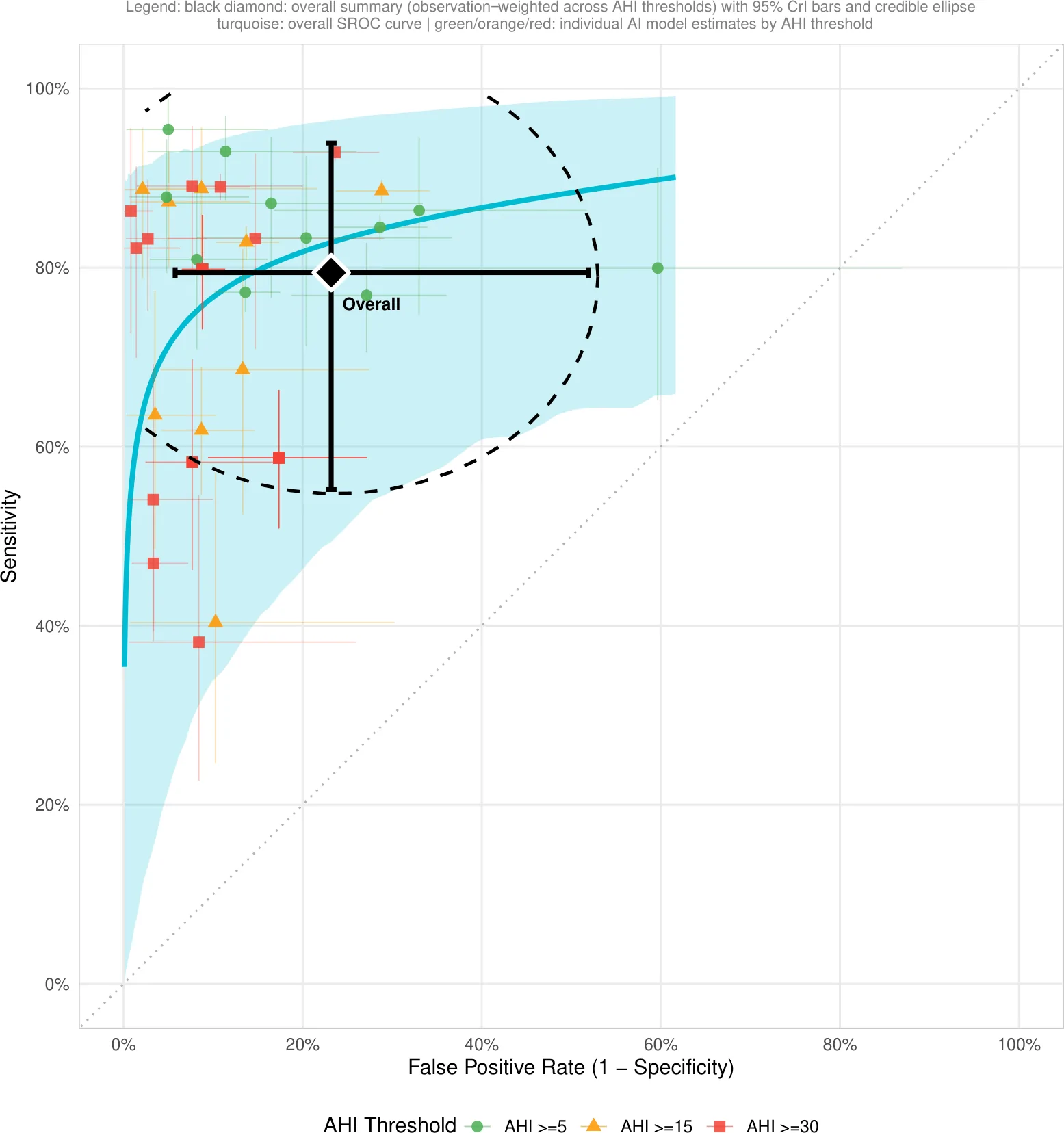
Summary receiver operating characteristic (SROC) curve for Bayesian 2-level bivariate model. AHI: apnea-hypopnea index; CrI: credible interval.

PPG-based AI models demonstrated a pooled sensitivity of 79.6% (95% CrI 55.5%‐93.8%) and a specificity of 76.5% (95% CrI 48.2%‐94.0%; Figure 2), compared to traditional diagnostic methods. These estimates indicated moderate diagnostic performance, although the wide CrI suggests substantial between-study variability. The SROC curve is presented in Figure 3. Model diagnostics indicated good convergence (all R̂≤1.01) with adequate effective sample sizes.

In a clinical diagnostic context, the application of Bayes theorem allows for the interpretation of findings related to OSA prevalence. When considering a pretest probability, or baseline population prevalence of OSA, of approximately 15% (Iceland, Indonesia, and the United Arab Emirates), the results indicate that a positive diagnostic test result would yield an average posttest probability of 37.4% (95% CrI 15.9%‐73.4%). For a population prevalence of 30% (United States, United Kingdom, and Samoa), the average posttest probability would be 59.2% (95% CrI 31.5%‐87.0%). For regions with a population prevalence of 60% (Singapore, Switzerland, and France), a positive test would yield a posttest probability of 83.6% (95% CrI 61.6%‐95.9%). Conversely, a negative test result would correspond to an average posttest probability of 4.5% (95% CrI 1.2%‐14.0%), 10.3% (95% CrI 2.8%‐28.3%), or 28.6% (95% CrI 9.0%‐58.1%) for the same prevalence categories. Furthermore, in instances where a patient’s pretest probability of OSA exceeds the regional prevalence, such as when the patient exhibits symptoms or signs indicative of OSA, the posttest probability is likely to be even higher.

### Meta-Regression

Meta-regression analysis was performed for OSA severity based on AHI thresholds, AI model type, type of validation method, OSA prevalence, and type of sleep device.

The specificities of detecting OSA on AI-trained PPG increased with categorical OSA severity. Pooled specificities at AHI thresholds of ≥5, ≥15, and ≥30 were 63.6%, 81.8%, and 85.1%, respectively. This trend was supported by positive logit-scale coefficients (≥15 vs ≥5: 0.94, 95% CrI 0.02‐1.83; ≥30 vs ≥5: 1.18, 95% CrI 0.15‐2.08). However, sensitivity appeared to decrease modestly with increasing categorical OSA severity. Pooled sensitivities at AHI thresholds of ≥5, ≥15, and ≥30 were 87.2%, 79.7%, and 76.7%, respectively, although the corresponding coefficients were imprecise and compatible with little or no difference (≥15 vs ≥5: −0.55, 95% CrI −1.24 to 0.11; ≥30 vs ≥5: −0.56, 95% CrI −1.22 to 0.12; Figure S1A in Multimedia Appendix 1).

Further meta-regression of AI model type demonstrated that deep learning models, such as convolutional neural networks, were associated with higher specificity of 82.9%, compared with other traditional machine learning models such as decision trees, hyperdimensional models, linear regression, logistic regression, local binary patterns, support vector machines, and combinations of these methods, which achieved a pooled specificity of 63.6%. This was supported by a positive coefficient (1.02, 95% CrI 0.02‐2.07). There was no clear difference in sensitivity (−0.02, 95% CrI −0.56 to 0.52; Figure S1B in Multimedia Appendix 1).

Additionally, studies that used external validation were associated with better sensitivity of 87.2% compared to studies using cross-validation (80.1%; −0.53, 95% CrI −0.80 to −0.27), whereas the comparison with studies adopting random split approaches was less certain (78.6%; −0.62, 95% CrI −1.58 to 0.41). For specificity, cross-validation was associated with higher estimates compared to external validation (0.87, 95% CrI 0.44‐1.30), whereas its comparison with the estimate for random split approaches was more uncertain (0.66, 95% CrI −0.63 to 1.91; Figure S1C in Multimedia Appendix 1).

The type of device used (wearable smart devices vs clinical oximeters; Figure S1D in Multimedia Appendix 1) and the prevalence of OSA (Figure S2 in Multimedia Appendix 1) were not clearly associated with sensitivity or specificity, with all corresponding 95% CrIs including the null value.

### Publication Bias

While visual inspection of funnel plots suggested possible asymmetry (Figure S3 in Multimedia Appendix 1), Deeks test did not suggest publication bias (intercept=−1.53; *P*=.14). Further sensitivity analyses on the SROC curve outlined no clinically significant publication bias. When evaluating other distinct mechanisms of publication bias, each with varying probabilities of unpublished studies of up to 60%, the SROC curve (Figure S4 in Multimedia Appendix 1) remained nearly unchanged. This suggests that even if a majority of studies had remained unpublished, the overall conclusions of the meta-analysis would not have been affected.

### Quality of Evidence

The overall quality of evidence per outcome assessed using GRADE was moderate (Table S5 in Multimedia Appendix 1).

## Discussion

### Principal Findings

This meta-analysis found that AI models trained on PPG recordings demonstrated favorable accuracy in OSA diagnosis, achieving a good sensitivity of 79.6% and specificity of 76.5%, compared to traditional diagnostic methods. To the best of the authors’ knowledge, this is the most comprehensive pooled analysis to date on the diagnostic accuracy of AI algorithms using PPG for OSA diagnosis.

### Comparison to Prior Work

This study builds on preexisting literature surrounding the accuracy of using PPG-based AI models in OSA diagnosis. A prior descriptive systematic review by Osa-Sanchez et al [36] found that integrating wearable devices with AI algorithms shows strong promise for real-time, portable, and early detection of OSA. However, a pooled meta-analysis was not performed in their prior study. Separately, Abd-Alrazaq et al [37] conducted a systematic review and meta-analysis of 38 studies to evaluate the diagnostic accuracy of wearable AI in detecting apneic events in respiration and OSA. They found that AI-powered wearable devices achieved a mean accuracy of 86.9%, with good sensitivity of 93.8% but lower specificity of 75.2% in detecting OSA. The current study provides a more detailed evaluation of existing evidence by incorporating additional sensitivity, subgroup, and publication bias analyses. A Bayesian hierarchical framework was employed to evaluate diagnostic accuracy outcomes holistically under various scenarios.

Unlike traditional approaches or simple machine learning models, deep learning relies on multilayered neural networks to capture more complex patterns within data [38]. Prior research has demonstrated that deep learning significantly improves the detection of subtle physiological changes associated with OSA by enabling the automatic extraction of intricate, high-dimensional features directly from PPG recordings [39]. This ability to autonomously identify relevant patterns in large datasets allows deep learning models to capture complex relationships that may not be immediately apparent through conventional methods.

Moreover, the automation offered by deep learning models presents significant logistical advantages over traditional diagnostic methods by reducing the need for manual intervention and expert oversight [40]. These AI-driven approaches can streamline diagnostic processes, improving efficiency and speed. Crucially, this provides an opportunity for reliable OSA diagnosis in low-resource or cost-constrained settings, where traditional methods may be prohibitively expensive or logistically challenging. WatchPAT (ZOLL Itamar) is an established HSAT modality for OSA diagnosis, particularly among patients with a high pretest probability of disease [41]. Given the encouraging diagnostic performance observed for PPG-based AI models in this review, these approaches warrant future direct comparison with established HSAT platforms to evaluate whether they offer comparable or incremental diagnostic value in clinical practice.

### Interpretation of Findings

Through meta-regression analysis, it was observed that the specificity of AI models trained on PPG data for detecting OSA improves as the severity of OSA increases. With increasing severity of OSA, oxygen desaturations become more frequent, deeper, and longer lasting during apneic episodes [42]. These alterations in blood oxygen saturation within the microvascular tissue bed are more pronounced and may be more easily detected by PPG technology, thereby improving between-group separability, which contributes to higher specificity. Consequently, AI-based systems have the potential to be more effective in diagnosing severe cases of OSA correctly, where the physiological markers are clearer and more detectable. Conversely, this may also imply that for AI models to achieve accurate detection across the entire spectrum of OSA severity, further refinement is needed, particularly to improve their ability to identify mild cases.

However, the sensitivity of OSA detection appears to decrease with greater categorical OSA severity. AI-assisted PPG models may produce a higher rate of false negatives in more severe cases. They may be more accurate at distinguishing individuals with and without OSA than at distinguishing the severity of OSA. Severe OSA is inherently heterogeneous, with variable phenotypes, variable event morphology, and substantial interindividual variability in desaturation dynamics, autonomic response, vascular tone, and comorbid cardiovascular conditions, which may influence PPG detection of OSA [43,44]. The divergent trends in specificity and sensitivity across severity strata reflect the complex dynamics of harnessing AI to diagnose OSA using PPG. Future research may help clarify the relationship between OSA severity and diagnostic accuracy.

Additionally, meta-regression analysis showed that deep learning achieved superior specificity compared to traditional machine learning models. This finding suggests that the ability of deep learning to autonomously identify complex patterns from PPG signals enhances its detection of subtle physiological alterations associated with OSA [45]. Separately, traditional machine learning may depend on manually engineered features, which may limit its sensitivity. While no evidence of a difference in sensitivity was observed, higher specificity was observed for deep learning models, which highlights the potential utility of deep learning models in OSA diagnosis.

Validation strategy may influence reported diagnostic performance, with external validation associated with greater sensitivity and cross-validation with greater estimates for specificity, although comparisons involving random split approaches remained uncertain. Nonetheless, the limited number of studies using each method of validation may preclude conclusions regarding an optimal validation strategy. Future studies using consistent validation frameworks across comparable datasets may help clarify the impact of validation strategy on reported diagnostic accuracy.

### Limitations

This study has several limitations. First, the underrepresentation of certain geographic groups in existing datasets may limit the generalizability of the findings. Second, the relatively small number of included studies, which typically feature small sample sizes, may introduce biases, as outliers from these small sample sizes could disproportionately affect the pooled results. Potential bias in preexisting studies may be driven by the use of convenience samples from sleep clinics, which may limit the applicability of existing studies. Third, publication bias remains a potential issue, as there is a possibility of missing relevant studies. Fourth, there was insufficient evidence to examine how BMI may influence the diagnostic accuracy outcome. High BMI is a recognized risk factor for OSA and is associated with greater disease severity [46]. The accuracy of diagnostic methods may be influenced by a patient’s BMI, and a thorough understanding of the relationship between BMI and PPG-based AI models may help guide clinical decision-making. Fifth, while all studies used validation set testing, some relied on internal test sets; external test sets are preferred for evaluating AI algorithms, as they better assess performance when applied to data from different sources [47]. Future research should focus on deep learning and incorporate prospective studies to enhance the feasibility and accessibility of this approach. Additionally, further studies are necessary to interpret these findings in a broader context and to optimize the diagnostic performance of AI-driven models in OSA diagnosis. Sixth, while the original PROSPERO registration outlined a broader review of AI modalities for OSA diagnosis, this manuscript focuses specifically on PPG due to the volume and diversity of available studies. Nonetheless, no alterations were made to the analytical methods, which remained consistent with the protocol. Seventh, heterogeneity was observed in pooled analyses, which was potentially driven by threshold effects, variation in disease severity, differences in study design, and evaluation frameworks. While additional meta-regression analyses were performed, results should be interpreted in the context of potential variability.

### Future Directions

It is essential for future research to carefully evaluate the trade-offs involved in incorporating additional signal channels into AI-driven PPG systems, particularly in the context of the costs and practicalities associated with diagnosing OSA. Although this study did not include a formal cost-benefit analysis, it highlights several critical factors for consideration: balancing diagnostic accuracy against signal complexity, assessing both hardware and operational costs, promoting user compliance and comfort, and addressing the interpretability and generalizability of AI models. Future research should also concentrate on deep learning techniques to enhance the feasibility and accessibility of this approach, particularly within primary care settings. The inclusion of prospective, real-world data may also enhance the credibility of AI-assisted OSA diagnosis.

### Conclusions

This meta-analysis suggests that AI models trained on PPG data may demonstrate reasonable diagnostic accuracy. Considering the logistical and economic challenges associated with current sleep testing, AI-driven models could serve as a cost-effective screening tool, pending further validation.

## Supplementary material

10.2196/78718 Multimedia Appendix 1 Supplementary Materials.

10.2196/78718 Checklist 1 PRISMA checklist.

## Abbreviations

AHI: apnea-hypopnea index
CrI: credible interval
GRADE: Grading of Recommendations, Assessment, Development and Evaluations
HSAT: home sleep apnea testing
OSA: obstructive sleep apnea
PPG: photoplethysmography
PRISMA: Preferred Reporting Items for Systematic Reviews and Meta-Analyses
PSG: polysomnography
QUADAS-2: Quality Assessment of Diagnostic Accuracy Studies-2
SROC: summary receiver operating characteristic

## References

[1] Gottlieb DJ, Punjabi NM (2020). Diagnosis and management of obstructive sleep apnea: a review. JAMA.

[2] Benjafield AV, Ayas NT, Eastwood PR (2019). Estimation of the global prevalence and burden of obstructive sleep apnoea: a literature-based analysis. Lancet Respir Med.

[3] Kapur V, Strohl KP, Redline S, Iber C, O’Connor G, Nieto J (2002). Underdiagnosis of sleep apnea syndrome in U.S. communities. Sleep Breath.

[4] Marino M, Li Y, Rueschman MN (2013). Measuring sleep: accuracy, sensitivity, and specificity of wrist actigraphy compared to polysomnography. Sleep.

[5] Markun LC, Sampat A (2020). Clinician-focused overview and developments in polysomnography. Curr Sleep Med Rep.

[6] Bazoukis G, Bollepalli SC, Chung CT (2023). Application of artificial intelligence in the diagnosis of sleep apnea. J Clin Sleep Med.

[7] Leong ZH, Loh SRH, Leow LC, Ong TH, Toh ST (2025). A machine learning approach for the diagnosis of obstructive sleep apnoea using oximetry, demographic and anthropometric data. Singapore Med J.

[8] Tan BKJ, Gao EY, Tan NKW (2025). Machine listening for OSA diagnosis: a Bayesian meta-analysis. Chest.

[9] Gao EY, Hao Y, Tan NKW (2025). Awake speech recordings for machine learning diagnosis of obstructive sleep apnea: a Bayesian meta-analysis. J Clin Sleep Med.

[10] Gao EY, Tan BKJ, Tan NKW (2024). Artificial intelligence facial recognition of obstructive sleep apnea: a Bayesian meta-analysis. Sleep Breath.

[11] Hao Y, Tan NKW, Gao EY (2025). Electrocardiogram heart rate variability for machine learning diagnosis of obstructive sleep apnoea: a Bayesian meta-analysis. Sleep Breath.

[12] Allen J (2007). Photoplethysmography and its application in clinical physiological measurement. Physiol Meas.

[13] Wei K, Zou L, Liu G, Wang C (2023). MS-Net: Sleep apnea detection in PPG using multi-scale block and shadow module one-dimensional convolutional neural network. Comput Biol Med.

[14] Papini GB, Fonseca P, van Gilst MM, Bergmans JWM, Vullings R, Overeem S (2020). Wearable monitoring of sleep-disordered breathing: estimation of the apnea-hypopnea index using wrist-worn reflective photoplethysmography. Sci Rep.

[15] Haug CJ, Drazen JM (2023). Artificial intelligence and machine learning in clinical medicine, 2023. N Engl J Med.

[16] Page MJ, McKenzie JE, Bossuyt PM (2021). The PRISMA 2020 statement: an updated guideline for reporting systematic reviews. BMJ.

[17] Cerullo E, Sutton AJ, Jones HE, Wu O, Quinn TJ, Cooper NJ (2023). MetaBayesDTA: codeless Bayesian meta-analysis of test accuracy, with or without a gold standard. BMC Med Res Methodol.

[18] Mizutani S, Zhou Y, Tian YS, Takagi T, Ohkubo T, Hattori S (2023). DTAmetasa: An R shiny application for meta-analysis of diagnostic test accuracy and sensitivity analysis of publication bias. Res Synth Methods.

[19] Freeman SC, Kerby CR, Patel A, Cooper NJ, Quinn T, Sutton AJ (2019). Development of an interactive web-based tool to conduct and interrogate meta-analysis of diagnostic test accuracy studies: MetaDTA. BMC Med Res Methodol.

[20] Patel A, Cooper N, Freeman S, Sutton A (2021). Graphical enhancements to summary receiver operating characteristic plots to facilitate the analysis and reporting of meta-analysis of diagnostic test accuracy data. Res Synth Methods.

[21] Deeks JJ, Macaskill P, Irwig L (2005). The performance of tests of publication bias and other sample size effects in systematic reviews of diagnostic test accuracy was assessed. J Clin Epidemiol.

[22] Whiting PF, Rutjes AWS, Westwood ME (2011). QUADAS-2: a revised tool for the quality assessment of diagnostic accuracy studies. Ann Intern Med.

[23] Guyatt GH, Oxman AD, Vist GE (2008). GRADE: an emerging consensus on rating quality of evidence and strength of recommendations. BMJ.

[24] Schünemann HJ, Mustafa RA, Brozek J (2020). GRADE guidelines: 21 part 2. test accuracy: inconsistency, imprecision, publication bias, and other domains for rating the certainty of evidence and presenting it in evidence profiles and summary of findings tables. J Clin Epidemiol.

[25] Attia S, Oksenberg A, Levy J (2026). Clinical validation of artificial intelligence algorithms for the diagnosis of adult obstructive sleep apnea and sleep staging from oximetry and photoplethysmography-SleepAI. J Sleep Res.

[26] Behar J, Roebuck A, Shahid M (2015). SleepAp: an automated obstructive sleep apnoea screening application for smartphones. IEEE J Biomed Health Inform.

[27] Browne SH, Vaida F, Umlauf A, Kim J, DeYoung P, Owens RL (2024). Performance of a commercial smart watch compared to polysomnography reference for overnight continuous oximetry measurement and sleep apnea evaluation. J Clin Sleep Med.

[28] Chen T, Zhang J, Xu Z (2024). Energy-efficient sleep apnea detection using a hyperdimensional computing framework based on wearable bracelet photoplethysmography. IEEE Trans Biomed Eng.

[29] Chen KW, Tseng CH, Lee HC, Liu WT, Chou KT, Wu HT (2025). Validation of a fingertip home sleep apnea testing system using deep learning AI and a temporal event localization analysis. Sleep.

[30] Guo H, Wu H, Xia J OSAHS detection capabilities of ringconn smart ring: a feasibility study. https://ieeexplore.ieee.org/document/10595903.

[31] Kim D, Han JY, Jung H (2025). AI‑enhanced smartwatch AHI estimation and AI-scored polysomnography for obstructive sleep apnea: real‑world validation. Nat Sci Sleep.

[32] Liang Z (2024). Developing probabilistic ensemble machine learning models for home-based sleep apnea screening using overnight SpO2 data at varying data granularity. Sleep Breath.

[33] Shen Q, Yang X, Zou L, Wei K, Wang C, Liu G (2022). Multitask residual shrinkage convolutional neural network for sleep apnea detection based on wearable bracelet photoplethysmography. IEEE Internet Things J.

[34] Tirachaimongkol V, Banhiran W, Chotinaiwattarakul W (2025). Belun Sleep Platform versus in-lab polysomnography for obstructive sleep apnea diagnosis. Sleep Breath.

[35] Zou L, Liu G (2024). Multiscale bidirectional temporal convolutional network for sleep apnea detection based on wearable photoplethysmography bracelet. IEEE J Biomed Health Inform.

[36] Osa-Sanchez A, Ramos-Martinez-de-Soria J, Mendez-Zorrilla A, Ruiz IO, Garcia-Zapirain B (2025). Wearable sensors and artificial intelligence for sleep apnea detection: a systematic review. J Med Syst.

[37] Abd-Alrazaq A, Aslam H, AlSaad R (2024). Detection of sleep apnea using wearable AI: systematic review and meta-analysis. J Med Internet Res.

[38] Sarker IH (2021). Deep learning: a comprehensive overview on techniques, taxonomy, applications and research directions. SN Comput Sci.

[39] Park MJ, Choi JH, Kim SY, Ha TK (2024). A deep learning algorithm model to automatically score and grade obstructive sleep apnea in adult polysomnography. Digit Health.

[40] Krishnan G, Singh S, Pathania M (2023). Artificial intelligence in clinical medicine: catalyzing a sustainable global healthcare paradigm. Front Artif Intell.

[41] Ioachimescu OC, Allam JS, Samarghandi A (2020). Performance of peripheral arterial tonometry-based testing for the diagnosis of obstructive sleep apnea in a large sleep clinic cohort. J Clin Sleep Med.

[42] Lv R, Liu X, Zhang Y (2023). Pathophysiological mechanisms and therapeutic approaches in obstructive sleep apnea syndrome. Signal Transduct Target Ther.

[43] Redline S, Azarbarzin A, Peker Y (2023). Obstructive sleep apnoea heterogeneity and cardiovascular disease. Nat Rev Cardiol.

[44] Xie A, Bedekar A, Skatrud JB, Teodorescu M, Gong Y, Dempsey JA (2011). The heterogeneity of obstructive sleep apnea (predominant obstructive vs pure obstructive apnea). Sleep.

[45] Janiesch C, Zschech P, Heinrich K (2021). Machine learning and deep learning. Electron Markets.

[46] Romero-Corral A, Caples SM, Lopez-Jimenez F, Somers VK (2010). Interactions between obesity and obstructive sleep apnea: implications for treatment. Chest.

[47] Cabitza F, Campagner A, Soares F (2021). The importance of being external. methodological insights for the external validation of machine learning models in medicine. Comput Methods Programs Biomed.

