# Supplementary material for: Accuracy of Artificial Intelligence in Diagnosing Obstructive Sleep Apnea Using Photoplethysmography: Systematic Review and Meta-Analysis
Source: J Med Internet Res. 2026 Sep 25;28:e78718. doi: 10.2196/78718 (PMC13614509; doi:10.2196/78718)
Supplement: Supplementary file 1 [file jmir-v28-e78718-s001.docx]

**SUPPLEMENTARY METHODS**

**Search Strategy**

| Overall Search Strategy | (("sleep apnea" OR "sleep apnoea" OR "nocturnal hypoxia" OR "nocturnal hypoxaemia" OR "nocturnal hypoxemia" OR "sleep disordered breathing") AND ("artificial intelligence" OR "machine learning" OR "deep learning" OR "logistic regression" OR "support vector machine" OR "neural network" OR "classification tree" OR "regression tree" OR "probability tree" OR "nearest neighbour" OR "nearest neighbor" OR "fuzzy logic" OR "naive bayes" OR "genetic algorithm" OR "multilayer perceptron" OR "random forest" OR "lasso regression" OR "kernel regression" OR "elastic net" OR "generative model" OR "generative adversarial network" OR "large language model") AND (diagnosis OR diagnose OR detect OR detection OR identify OR identification OR severity OR classify OR classification)) |
| --- | --- |
| PubMed | (  "sleep apnea"[All Fields]  OR "sleep apnoea"[All Fields]  OR "nocturnal hypoxia"[All Fields]  OR "nocturnal hypoxaemia"[All Fields]  OR "nocturnal hypoxemia"[All Fields]  OR "sleep disordered breathing"[All Fields]  )  AND  (  "artificial intelligence"[All Fields]  OR "machine learning"[All Fields]  OR "deep learning"[All Fields]  OR "logistic regression"[All Fields]  OR "support vector machine"[All Fields]  OR "neural network"[All Fields]  OR "classification tree"[All Fields]  OR "regression tree"[All Fields]  OR "probability tree"[All Fields]  OR "nearest neighbour"[All Fields]  OR "nearest neighbor"[All Fields]  OR "fuzzy logic"[All Fields]  OR "naive bayes"[All Fields]  OR "genetic algorithm"[All Fields]  OR "multilayer perceptron"[All Fields]  OR "random forest"[All Fields]  OR "lasso regression"[All Fields]  OR "kernel regression"[All Fields]  OR "elastic net"[All Fields]  OR "generative model"[All Fields]  OR "generative adversarial network"[All Fields]  OR "large language model"[All Fields]  )  AND  (  "diagnosable"[All Fields]  OR "diagnosi"[All Fields]  OR "diagnosis"[All Fields]  OR "diagnosis"[MeSH Terms]  OR "diagnosis"[MeSH Subheading]  OR "diagnose"[All Fields]  OR "diagnosed"[All Fields]  OR "diagnoses"[All Fields]  OR "diagnosing"[All Fields]  OR "detect"[All Fields]  OR "detected"[All Fields]  OR "detecting"[All Fields]  OR "detection"[All Fields]  OR "detections"[All Fields]  OR "identifiable"[All Fields]  OR "identifiably"[All Fields]  OR "identifie"[All Fields]  OR "identified"[All Fields]  OR "identifier"[All Fields]  OR "identifiers"[All Fields]  OR "identifies"[All Fields]  OR "identify"[All Fields]  OR "identifying"[All Fields]  OR "identifed"[All Fields]  OR "identification"[All Fields]  OR "identifications"[All Fields]  OR "identification, psychological"[MeSH Terms]  OR "psychological identification"[All Fields]  OR "severe"[All Fields]  OR "severely"[All Fields]  OR "severity"[All Fields]  OR "severities"[All Fields]  OR "classifiable"[All Fields]  OR "classification"[All Fields]  OR "classification"[MeSH Terms]  OR "classification"[MeSH Subheading]  OR "classified"[All Fields]  OR "classify"[All Fields]  OR "classifying"[All Fields]  OR "classifier"[All Fields]  OR "classifiers"[All Fields]  OR "classifies"[All Fields]  OR "classifications"[All Fields]  OR "classificator"[All Fields]  OR "classificators"[All Fields]  ) |
| Embase | ('sleep apnea'/exp OR 'sleep apnea' OR 'sleep apnoea'/exp OR 'sleep apnoea' OR 'nocturnal hypoxia'/exp OR 'nocturnal hypoxia' OR 'nocturnal hypoxaemia' OR 'nocturnal hypoxemia'/exp OR 'nocturnal hypoxemia' OR 'sleep disordered breathing'/exp OR 'sleep disordered breathing') AND ('artificial intelligence'/exp OR 'artificial intelligence' OR 'machine learning'/exp OR 'machine learning' OR 'deep learning'/exp OR 'deep learning' OR 'logistic regression'/exp OR 'logistic regression' OR 'support vector machine'/exp OR 'support vector machine' OR 'neural network'/exp OR 'neural network' OR 'classification tree'/exp OR 'classification tree' OR 'regression tree'/exp OR 'regression tree' OR 'probability tree' OR 'nearest neighbour' OR 'nearest neighbor' OR 'fuzzy logic'/exp OR 'fuzzy logic' OR 'naive bayes'/exp OR 'naive bayes' OR 'genetic algorithm'/exp OR 'genetic algorithm' OR 'multilayer perceptron'/exp OR 'multilayer perceptron' OR 'random forest'/exp OR 'random forest' OR 'lasso regression'/exp OR 'lasso regression' OR 'kernel regression'/exp OR 'kernel regression' OR 'elastic net'/exp OR 'elastic net' OR 'generative model'/exp OR 'generative model' OR 'generative adversarial network'/exp OR 'generative adversarial network' OR 'large language model') AND ('diagnosis'/exp OR diagnosis OR diagnose OR detect OR 'detection'/exp OR detection OR identify OR 'identification'/exp OR identification OR 'severity'/exp OR severity OR classify OR 'classification'/exp OR classification) NOT [medline]/lim AND ([article]/lim OR [article in press]/lim) AND [english]/lim |
| Scopus | ("sleep apnea" OR "sleep apnoea" OR "nocturnal hypoxia" OR "nocturnal hypoxaemia" OR "nocturnal hypoxemia" OR "sleep disordered breathing" ) AND ( "artificial intelligence" OR "machine learning" OR "deep learning" OR "logistic regression" OR "support vector machine" OR "neural network" OR "classification tree" OR "regression tree" OR "probability tree" OR "nearest neighbour" OR "nearest neighbor" OR "fuzzy logic" OR "naive bayes" OR "genetic algorithm" OR "multilayer perceptron" OR "random forest" OR "lasso regression" OR "kernel regression" OR "elastic net" OR "generative model" OR "generative adversarial network" OR "large language model" ) AND ( diagnosis OR diagnose OR detect OR detection OR identify OR identification OR severity OR classify OR classification ) AND NOT INDEX ( medline ) AND ( LIMIT-TO ( DOCTYPE , "ar" ) ) AND ( LIMIT-TO ( LANGUAGE , "English" ) ) AND ( LIMIT-TO ( EXACTKEYWORD , "Human" ) ) |
| Web of Science | ("sleep apnea" OR "sleep apnoea" OR "nocturnal hypoxia" OR "nocturnal hypoxaemia" OR "nocturnal hypoxemia" OR "sleep disordered breathing") AND ("artificial intelligence" OR "machine learning" OR "deep learning" OR "logistic regression" OR "support vector machine" OR "neural network" OR "classification tree" OR "regression tree" OR "probability tree" OR "nearest neighbour" OR "nearest neighbor" OR "fuzzy logic" OR "naive bayes" OR "genetic algorithm" OR "multilayer perceptron" OR "random forest" OR "lasso regression" OR "kernel regression" OR "elastic net" OR "generative model" OR "generative adversarial network" OR "large language model") AND (diagnosis OR diagnose OR detect OR detection OR identify OR identification OR severity OR classify OR classification)  Limits Applies: Article, English Language |
| IEEE Xplore | ("sleep apnea" OR "nocturnal hypoxemia" OR "sleep disordered breathing") AND ("artificial intelligence" OR "machine learning" OR "deep learning" OR "logistic regression" OR "support vector machine" OR "neural network" OR "classification tree" OR "regression tree" OR "probability tree" OR "nearest neighbor" OR "random forest" OR "generative model" OR "generative adversarial network" OR "large language model") AND (diagnos* OR detect* OR identif* OR severity OR classif*) |

# **ONLINE-ONLY TABLES**

**Table S1: Inclusion Criteria**

| **Category** | **Inclusion Criteria** |
| --- | --- |
| Population | Adults Aged ≥ 18 Years Old |
| Intervention/Exposure | Diagnosis and Classification of Obstructive Sleep Apnea Using Artificial Intelligence, Including Both Traditional and Regression Techniques and Machine Learning Models, Using Photoplethysmography |
| Comparators | Diagnosis and Classification of Obstructive Sleep Apnea Using Traditional Apnea-Hypopnea Index from Overnight Polysomnography or Home Sleep Apnea Testing |
| Outcomes | Accuracy of Artificial Intelligence for The Diagnosis and Classification of Obstructive Sleep Apnea |
| Study Designs | Observational Studies |

**Table S2: Exclusion Criteria**

| **Number** | **Exclusion Criteria** |
| --- | --- |
| 1 | Case Reports |
| 2 | Case Series |
| 3 | Reviews |
| 4 | Meta-Analyses |
| 5 | Letters |
| 6 | Conference Abstracts |
| 7 | Paediatric Studies |
| 8 | Animal Studies |
| 9 | Foreign Language Studies (Non-English) |
| 10 | Studies With Incomplete Data |
| 11 | Studies Focusing on Individual Apnoeic Events Without Patient-Level Classification |

**Table S3: Summary of Included Studies**

| **First Author and Year** | **Country** | **% Male** | **Mean**  **Age** | **Mean BMI** | **Sample Size** | **Sleep Study** | **Reference** | **AHI**  **Cut-Offs** | **OSA Prevalence** | **Primary Validation Method** | **Artificial Intelligence Model Type** | **Sleep**  **Device** |
| --- | --- | --- | --- | --- | --- | --- | --- | --- | --- | --- | --- | --- |
| Attia et al., 2026^25^ | Israel | 64.2 | 47.2 | 27.7 | 53 | PSG | PSG AHI >= 5  PSG AHI >= 15  PSG AHI >= 30 | 5  15  30 | 71.7 | External Validation | Deep Machine Learning | Wearable  Smart  Device |
| Behar et al., 2015^26^ | UK | 68.3 | 49.4 | 33.1 | 856 | HSAT | HSAT AHI >= 5 | 5 | 51.9 | Random-Split | Traditional Machine Learning – Support Vector Machine | Clinical Oximeter |
| Browne et al., 2024^27^ | USA | 66.7 | 49.5 | 28.1 | 51 | PSG | PSG AHI >= 5  PSG AHI >= 15  PSG AHI >= 30 | 5  15  30 | 58.8 | External Validation | Traditional Machine Learning - Logistic Regression | Wearable  Smart  Device |
| Chen et al., 2024^28^ | China | NR | NR | NR | 32 | PSG | PSG AHI >= 30 | 30 | 68.5 | Cross-Validation | Traditional Machine Learning – Logistic Regression, Decision Trees, Support Vector Machine, 1-Dimensional Block Local Binary Pattern, Hyperdimensional Computing, 1-Dimensional Block Local Binary Pattern + Hyperdimensional Computing (Proposed Model), Convolutional Neural Network | Wearable  Smart  Device |
| Chen et al., 2025^29^ | Taiwan | 74 | 49.3 | 26.9 | 331 | PSG | PSG AHI >= 5  PSG AHI >= 15  PSG AHI >= 30 | 5  15  30 | 81.5 | External Validation | Deep Machine Learning - Convolutional Neural Network | Wearable  Smart  Device |
| Guo et al., 2024^30^ | China | 71 | 52.2 | 26.0 | 58 | PSG | PSG AHI >= 5  PSG AHI >= 15  PSG AHI >= 30 | 5  15  30 | 58.6 | Random-Split | Deep Machine Learning - Convolutional Neural Network | Wearable  Smart  Device |
| Kim et al., 2025^31^ | South Korea | 71.7 | 42 | 24.9 | 53 | PSG | PSG AHI >= 5  PSG AHI >= 15  PSG AHI >= 30 | 5  15  30 | 67.8 | External Validation | Traditional Machine Learning | Wearable  Smart  Device |
| Liang, 2024 (Sleep Heart Health Study)^32^ | USA | 47.7 | 63.3 | 28.1 | 5667 | PSG | PSG AHI >= 5  PSG AHI >= 15  PSG AHI >= 30 | 5  15  30 | 82.8 | Cross-Validation | Traditional Machine Learning – Support Vector Machine, Logistic Regression, Light Gradient Boosting Machine | Wearable  Smart  Device |
| Liang, 2024 (Multi-Ethnic Study of Atherosclerosis)^32^ | USA | 46.4 | 69.4 | 28.7 | 2051 | PSG | PSG AHI >= 5  PSG AHI >= 15  PSG AHI >= 30 | 5  15  30 | 89.5 | External Validation | Traditional Machine Learning – Support Vector Machine, Logistic Regression, Light Gradient Boosting Machine | Wearable  Smart  Device |
| Papini et al., 2020^14^ | Netherlands | 62.9 | 48 | 27 | 502 | PSG | PSG AHI >= 5  PSG AHI >= 15  PSG AHI >= 30 | 5  15  30 | 63.5 | Random-Split | Traditional Machine Learning - Linear Regression | Wearable  Smart  Device |
| Shen et al., 2022^33^ | China | NR | NR | NR | 92 | PSG | PSG AHI >= 5 | 5 | 69.57 | Random-Split | Deep Machine Learning - Convolutional Neural Network | Wearable  Smart  Device |
| Tirachaimongkol et al., 2025^34^ | Thailand | 54.3 | 49.9 | NR | 35 | PSG | PSG AHI >= 5  PSG AHI >= 15  PSG AHI >= 30 | 5  15  30 | 80 | External Validation | Deep Machine Learning – Cloud-Based Neural Network Algorithm | Wearable  Smart  Device |
| Wei et al., 2023^13^ | China | 69.1 | 45.3 | NR | 110 | PSG | PSG AHI >= 30 | 30 | 22.7 | Cross-Validation | Deep Machine Learning - Convolutional Neural Network | Clinical Oximeter |
| Zou et al., 2024^35^ | China | NR | NR | NR | 92 | PSG | PSG AHI >= 30 | 30 | 29.3 | Cross-Validation | Deep Machine Learning - Convolutional Neural Network | Wearable  Smart  Device |

Legend: BMI: Body Mass Index; AHI: Apnoea Hypopnoea Index; OSA: Obstructive Sleep Apnea; PSG: Polysomnography; HSAT: Home Sleep Apnea Testing; UK: United Kingdom; USA: United States of America; NR: Not Reported

**Table S4: Risk of Bias Assessment Using The Quality Assessment of Diagnostic Accuracy Studies-2 (QUADAS-2) Tool**

| **Study** | **Risk of Bias** | | | | **Concerns Regarding Applicability** | | | **Overall Risk of Bias** |
| --- | --- | --- | --- | --- | --- | --- | --- | --- |
|  | **Patient Selection** | **Index Test** | **Reference Standard** | **Flow & Timing** | **Patient Selection** | **Index Test** | **Reference Standard** |  |
| Attia et al., 2026^25^ | Low | Low | Low | Low | Low | Low | Low | Low |
| Behar et al., 2015^26^ | Low | Low | Unclear | Low | Unclear | Low | Unclear | Unclear |
| Browne et al., 2024^27^ | Unclear | Low | Low | Low | Unclear | Low | Low | Unclear |
| Chen et al., 2024^28^ | Low | Unclear | Low | Low | Low | Low | Low | Unclear |
| Chen et al., 2025^29^ | Low | Low | Low | Low | Low | Low | Low | Low |
| Guo et al., 2024^30^ | Low | Unclear | Low | Low | Low | Low | Low | Unclear |
| Kim et al., 2025^31^ | Unclear | Low | Low | Unclear | Unclear | Low | Unclear | Unclear |
| Liang, 2024^32^ | Low | Low | Low | Low | Unclear | Low | Low | Unclear |
| Papini et al., 2020^14^ | Low | Low | Low | Low | Low | Low | Low | Low |
| Shen et al., 2022^33^ | Low | Low | Low | Low | Low | Low | Low | Low |
| Tirachaimongkol et al., 2025^34^ | Low | Low | Low | Low | Low | Low | Low | Low |
| Wei et al., 2023^13^ | Low | Low | Low | Low | Low | Low | Low | Low |
| Zou et al., 2024^35^ | Low | Low | Low | Low | Low | Low | Low | Low |

Whiting PF, Rutjes AW, Westwood ME, Mallett S, Deeks JJ, Reitsma JB, et al. QUADAS-2: a revised tool for the quality assessment of diagnostic accuracy studies. Ann Intern Med. 2011;155(8):529-36.

**Table S5: Certainty of Evidence Assessment Using The Grading of Recommendations Assessment, Development and Evaluation (GRADE) Framework**

| **Outcomes** | **Pooled Outcomes (95% CrI)** | **Number of Included Studies** | **Risk of Bias** | **Imprecision** | **Inconsistency** | **Indirectness** | **Publication Bias** | **Others** | **Quality of Evidence (GRADE)** |
| --- | --- | --- | --- | --- | --- | --- | --- | --- | --- |
| Sensitivity | 79.6% (95%CrI: 55.5-93.8%) | 13 | Serious | Serious | Not Serious | Not Serious | Not Serious | Dose Response Gradient* | ⊕⊕⊕⊖  Moderate |
| Specificity | 76.5% (95%CrI: 48.2-94.0%) | 13 | Serious | Serious | Not Serious | Not Serious | Not Serious | Dose Response Gradient* | ⊕⊕⊕⊖  Moderate |

The overall quality can be downgraded based on the following criteria: risk of bias, imprecision, inconsistency, indirectness and publication bias. The overall quality can be upgraded for the following reasons: a large effect size, a dose-response effect, or if all plausible confounders and other biases would increase confidence in the estimated effect. *Evidence was upgraded due to an observed categorical dose-response relationship between AHI thresholds and diagnostic accuracy, where greater AHI severity was associated with higher specificity and lower sensitivity.

Guyatt GH, Oxman AD, Vist GE, Kunz R, Falck-Ytter Y, Alonso-Coello P, et al. GRADE: an emerging consensus on rating quality of evidence and strength of recommendations. Bmj. 2008;336(7650):924-6.

Schünemann HJ, Mustafa RA, Brozek J, Steingart KR, Leeflang M, Murad MH, et al. GRADE guidelines: 21 part 2. Test accuracy: inconsistency, imprecision, publication bias, and other domains for rating the certainty of evidence and presenting it in evidence profiles and summary of findings tables. J Clin Epidemiol. 2020;122:142-52.

**ONLINE ONLY FIGURES**

**Figure S1: Summary Receiver Operating Characteristic Curves by Categorical Meta-Regression Covariates**

*Legend: Shaded Regions: 95% Credible Intervals. Points: Posterior Mean Operating Point +/- 95% Credible Interval. Dashed Line: Chance Diagonal*

**Figure S2: Summary Receiver Operating Characteristic Curve by Obstructive Sleep Apnea Prevalence**

*Legend: Solid Black Curve: Fitted Summary Receiver Operating Curve. Dashed Ellipse: 95% Credible Interval Around Summary Point. Black Circle: Pooled Estimate. Blue Circles: Individual Studies.*

**Figure S3: Contour-Enhanced Funnel Plot**

**Figure S4: Summary Receiver Operating Characteristic Curves for Publication Bias**
